# High Fat, High Sugar Diet and DJOS Bariatric Surgery Influence Plasma Levels of Fetuin-B, Growth Differentiation Factor-15, and Pentraxin 3 in Diet-Induced Obese Sprague–Dawley Rats

**DOI:** 10.3390/nu13103632

**Published:** 2021-10-17

**Authors:** Jakub Poloczek, Monika Tarnawska, Elżbieta Chełmecka, Piotr Łaszczyca, Janusz Gumprecht, Dominika Stygar

**Affiliations:** 1 Department of Rehabilitation, 3rd Specialist Hospital in Rybnik, Energetyków 46, 44-200 Rybnik, Poland; poloczek.jakub@gmail.com; 2 Department of Internal Medicine, Diabetology, and Nephrology, Faculty of Medical Sciences in Zabrze, Medical University of Silesia, Poniatowskiego 15, 40-055 Katowice, Poland; jgumprecht@sum.edu.pl; 3 Faculty of Natural Sciences, Institute of Biology, Biotechnology and Environmental Protection, University of Silesia in Katowice, Bankowa 9, 40-007 Katowice, Poland; monika.tarnawska@us.edu.pl (M.T.); piotr.laszczyca@us.edu.pl (P.Ł.); 4 Department of Statistics, Department of Instrumental Analysis, Faculty of Pharmaceutical Sciences in Sosnowiec, Medical University of Silesia, Poniatowskiego 15, 40-055 Katowice, Poland; echelmecka@sum.edu.pl; 5 Department of Physiology, Faculty of Medical Sciences in Zabrze, Medical University of Silesia, Poniatowskiego 15, 40-055 Katowice, Poland

**Keywords:** bariatric surgery, duodenojejunal omega switch (DJOS), fetuin-B, growth differentiation factor-15 (GDF-15), hepatokines, high fat sugar (HFS) diet, pentraxin 3 (PTX3)

## Abstract

The liver plays a central role in glucose and fatty acid metabolism and acts as an endocrine organ that secretes hepatokines with diverse systemic effects. The study aimed to examine the influence of duodenojejunal omega switch (DJOS) bariatric surgery in combination with different diets on glucose administration parameters and hepatokines levels. After 8 weeks on high fat, high sugar diet (HFS) or control diets (CD), Sprague–Dawley rats underwent DJOS or SHAM (control) surgery. For the next 8 weeks after the surgery, half of DJOS and SHAM-operated animals were kept on the same diet as before, and half had a diet change. The oral glucose tolerance test (OGTT) was performed three times: 8 weeks before and 4 and 8 weeks after surgery. Fetuin-B, growth differentiation factor-15 (GDF-15), pentraxin 3 (PTX3) plasma levels were analyzed. DJOS surgery had a beneficial effect on oral glucose tolerance test (OGTT) results and the area under the curve (AUC_OGTT_). The OGTT results depended on the time elapsed after the surgery, the type of diet used, the surgery performed, and the interaction between these factors. DJOS bariatric surgery reduced fetuin-B and GDF15 plasma levels. Interaction between the type of surgery performed and diet used influenced the fetuin-B and PTX-3 plasma levels. A dietary regime is essential to achieve therapeutic and clinical goals after bariatric surgery.

## 1. Introduction

Accumulation of hepatic fat is an often-encountered phenomenon in obese patients. It is a consequence of an increased influx of fatty acids to the liver and glucose-to-fat conversion in the liver [1]. The liver plays a central role in glucose and fatty acid metabolism and acts as an endocrine organ that secretes hepatokines that have diverse systemic effects [2,3].

Diminished physical activity accompanied by ready-to-eat energy-dense foods in the daily diet—the disreputable indicators of a westernized sedentary lifestyle filled with high fat and high carbohydrate dietary habits—are alarmingly common [4,5]. The increased use of a high fat/high sugar diet (HFS) lead the scientific community to postulate that HFS is related to the development of metabolic syndrome, type 2 *diabetes mellitus* (T2DM), and obesity during the last decades [6,7]. Bariatric surgeries are considered the most efficient procedures in terms of weight loss, especially the biliopancreatic diversion/duodenal switch groups followed by gastric bypass, gastroplasty, laparoscopic adjustable gastric banding. The estimated long-lasting effects of these treatments are similar up to about two years after the intervention [8]. Obesity and T2DM treatment with metabolic surgery influence the physiological role of hepatokines [8].

Fetuin-B is a hepatokine from a group of cysteine protease inhibitors occurring both in humans and rodents that negatively impact carbohydrate metabolism. Higher concentrations of fetuin-B were observed in people with non-alcoholic fatty liver disease (NAFLD) and T2DM [9]. Recently, fetuin-B was also reported as a risk factor of hepatic steatosis, disruption of glucose tolerance, and insulin homeostasis [9]. Growth differentiation factor-15 (GDF-15) belongs to the TGF-β (transforming growth factor beta) family. It is secreted in small amounts in most organs, including the liver [10,11]. GDF-15 has been found to control appetite, reduce body weight, increase thermogenesis, lipolysis and oxidative metabolism, improve insulin sensitivity and glucose tolerance [12] The diseases that involve organ damage (myocardial infarction, renal failure) or those which involve chronic inflammation (*diabetes mellitus*) elevate the GDF-15 concentrations [10,11]. Pentraxin 3 (PTX3) plays multiple roles, including a cardioprotective one [13,14,15]. It also acts as an anti-inflammatory protein [16]. As proved in experiments using PTX3 knock-out mice, it prevents the excessive expansion of the myocardial infarction area and the accumulation of aortic lesions [13,14]. Additionally, the circulatory level of pentraxin 3 (PTX3) is lower in obese individuals than in those with normal weight [17].

Since fetuin-B level significantly increases in individuals with hepatic disturbances, GDF-15, is produced by macrophages associated with a pro-inflammatory environment, and PTX3 is produced and released in response to primary inflammatory signals, such as IL-1 and TNF alpha [18], it seemed worth investigating the changes in their levels in the context of obesogenic diets and bariatric surgery. 

Studies using animal models that appropriately imitate all the aspects of human disease and present all major alterations of the illness are needed to develop successful strategies to treat metabolic syndrome. Since central obesity is a key factor in metabolic syndrome development, the diet-induced obesity (DIO) rodent models are frequently used to study pathways implied in metabolic syndrome development [6,19]. Currently, HFS diets are the most used models to mimic the so-called “western diet” [19,20].

In the presented study, we used a high fat/high sugar diet (HFS) to develop DIO in Sprague–Dawley rats to study the effects of duodenojejunal omega switch (DJOS) surgery on glucose administration parameters and hepatokines levels. In our experimental design, we applied the results from the human studies, showing that some patients failed to reduce their daily caloric intake after metabolic protocol. We assumed that, after surgery, patients might change their dietary preferences from a low-calorie diet to more energy-dense food and vice versa. The study design aimed to simulate the patients’ non-compliance and non-adherence to the post-surgery recommendations to reduce dietary fat and sugar intake. It also simulated the possibility that the patients might have switched from a regular to an HFS diet and from an HFS to a regular diet or adhered to a regular diet before and after the surgery.

## 2. Materials and Methods

All experimental procedures were approved by the Ethical Committee for Animal Experimentation of the Medical University of Silesia (Katowice, Poland) (58/2014) and all applicable institutional and national guidelines for the care and use of animals (Directive 2010/63/EU) were followed. We minimized the number of rats involved in the procedure according to “3Rs” for the humane treatment of animals [21]. All of the animals survived the experiment and post-surgery period (survival rate 100%).

### 2.1. Study Subject

The experiment used 56 male 7-weeks old Sprague–Dawley rats (Charles River Breeding Laboratories, Wilmington, MA, USA) with the initial weight ranging between 250 g and 275 g. Rats were housed in a plastic cage and fully controlled environment: 12/12 h of dark/light cycle, at 23 °C, with unrestricted access to food and water. 

The regular (control) diet (Provimi Kliba AG, Kaiseraugst, Switzerland) consisted of 10.8% sugar, 24% protein, 4.9% fat, 7% crude ashes, 4.7% crude fiber, and delivered 15.2 MJ/kg of energy. The high fat/high sugar (HFS) diet (ssniff^®^ EF R/M acc. D12451 (II) mod., ssniff Spezialdiäten GmbH, Soest, Germany) used for obesity induction consisted of 29.4% sugar, 20% protein, 45% fat, 35% carbohydrate, and delivered 22.0 MJ/kg of energy.

### 2.2. Study Design

**Figure 1:**
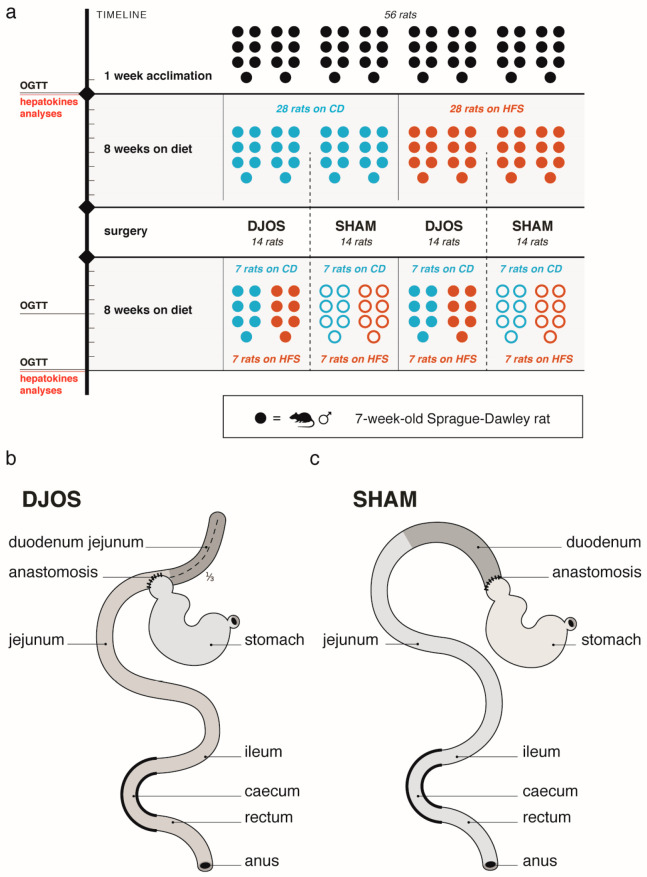
Study design: (**a**) experimental setup, (**b**) scheme of duodenojejunal omega switch (DJOS) surgery, (**c**) scheme of control (SHAM) surgery. Legend: CD—control diet, HFS—high fat, high sugar diet, DJOS—duodenojejunal omega switch, OGTT—oral glucose tolerance test, SHAM—control surgery.

After one week of acclimation, the animals were randomly assigned to the experimental groups (Figure 1a). The first part of the experiment assumed to maintain the rats on experimental diets: control diet (CD) (*n* = 28) or high fat/high sugar (HFS) (*n* = 28) for eight weeks. After this time, rats of both groups were randomly assigned to two subgroups that underwent a different type of surgery: control (SHAM) (*n* = 14) or duodenojejunal omega switch (DJOS) (*n* = 14). The second part of the experiment (after the surgery) assumed to maintain rats on the same diet as before the surgery (*n* = 7) (CD/SHAM/CD, CD/DJOS/CD, HFS/SHAM/HFS, HFS/DJOS/HFS) or on different diet as before the surgery (*n* = 7) (CD/SHAM/HFS, CD/DJOS/HFS, HFS/SHAM/CD, HFS/DJOS/CD) for further eight weeks. 

### 2.3. Experimental Procedures

#### 2.3.1. Control (SHAM) and Duodenojejunal Omega Switch (DJOS) Surgery

Control (SHAM) and duodenojejunal omega switch (DJOS) surgeries were performed according to the techniques described by Stygar et al. [22].

All animals were fasting the night before the surgery.

The anesthesia under spontaneous breathing was maintained with 2% isoflurane (AbbVie, Germany) and oxygen flow of 2 L/min. The analgesia was achieved with xylazine (5 mg/kg, ip; Xylapan, Vetoquinol Biovet, Puławy, Poland). The antibiotic prophylaxis was carried out using gentamicin (Gentamycin 40 mg/mL, Krka, Poland).

The abdominal access was achieved via a 3–4 cm midline incision. The duodenum was separated from the stomach, slightly distally to the pyloric sphincter, and new anastomosis was positioned at ⅓ of the small intestine total length. Then, the end-to-side anastomosis (duodeno-enterostomy) was performed to restore the bowel content passage and exclude the part of the small bowel and the duodenum (Figure 1b). The excluded part of the duodenum was stitched using PDS 6/0 (Ethicon, Cincinnati, OH, USA). The mesentery was also closed with PDS 6/0.

During SHAM surgery, the enterotomies and reanastomoses of the gastric tract were performed at the sites analogous for DJOS surgery, so the intestinal food passage was restored (Figure 1c).

The analgesia in the post-operative period was achieved using carprofen (4 mg/kg, sc; Rimadyl, Pfizer, Zürich, Switzerland) for three consecutive days.

#### 2.3.2. Oral Glucose Tolerance Test (OGTT) and Blood Collection

During the experiment, the glucose metabolism was assessed at three time points: 8 weeks before the surgery and 4 and 8 weeks after the surgery. 

The oral glucose tolerance test (OGTT) was performed under full sedation. The anesthesia was induced and maintained using isoflurane 2% and oxygen flow at a 2 L/min breathing rate. The OGTT started with the placement of an orogastric tube (central venous catheter, Arrow International Inc., Cleveland, OH, USA) and administration of 40% glucose solution at a 1.5 g/kg dosage. The glycemia was measured via tail snip at 0, 10, 30, 60, 90, and 120 min using a glucometer (Ascensia Elite, Bayer Corp., Langenfeld (Rheinland), Germany). 

Eight weeks after the surgery, 700 µL of blood was collected for hepatokines analysis. The blood was drawn from the right tail vein cannulated with the 26-gauge cannula at 0 and 30 min of oral glucose administration. The blood was stored using tubes containing 10 μL (7.2 mg) EDTA (Sigma-Aldrich, Burlington, MA, USA) and 4 μL dipeptidyl peptidase-4 inhibitor (DPP-4) (DRG Instruments, Marburg, Germany). The blood was centrifuged at 4000 rpm for 10 min at 4 °C. The collected plasma samples were snap-frozen in liquid nitrogen and stored at −80 °C until analyses. 

#### 2.3.3. Hepatokines Analysis

Fetuin-B (cat. no SEB860Ra), GDF-15 (cat. no SEC034Ra), pentraxin 3 (cat. no SEK411Ra) plasma concentrations were measured in duplicates using the rat enzyme-linked immunosorbent assay ELISA Kit (Wuhan USCN Business Co., Caidian Qu, Wuhan Shi, Hubei Sheng, China). The minimum detectable dose that the selected analytical kit could reliably measure was 13.1 ng/mL for fetuin-B, less than 6.2 pg/mL for GDF-15, and 13.6 pg/mL for pentraxin 3. The detection range of the used ELISA kits was 31.2–2000 ng/mL for fetuin-B, 15.6–1000 pg/mL for GDF-15, and 31.2–2000 pg/mL for pentraxin 3. 

### 2.4. Statistical Analysis

Statistical analysis was performed using Statistica 13.0 data analysis software system (TIBCO Software Inc., Palo Alto, CA, USA).

Statistical significance was set at a *p* < 0.05. Interval data were expressed as mean value ± standard deviation in the case of a normal distribution or as median (lower–upper quartile range) in the case of data with skewed or non-normal distribution. The distribution of variables was evaluated by the Shapiro–Wilk test and the quantile–quantile plot. The homogeneity of variances was assessed by the Levene test. The two-way parametric ANOVA with post hoc contrast analysis was used for data comparison. 

## 3. Results

### 3.1. Oral Glucose Tolerance Test (OGTT)

The results of the oral glucose tolerance test (OGTT) of Sprague–Dawley rats subjected to control (SHAM) and duodenojejunal omega switch (DJOS) surgery and different dietary regimes performed 8 weeks before and 4 and 8 weeks after the surgery are presented in Table 1.

The analysis of the OGTT results showed that the effect of time was statistically significant at all time points tested, regardless of the type of performed surgery and applied diet. A significant interaction between the time and diet was found for the results of OGTT carried out at 4 and 8 weeks after the surgery—the OGGT time profiles in individual dietary groups differed significantly among themselves depending on the type of dietary regime used. Interaction between the time, diet, and type of surgery was observed for the results of the OGGT at 4 and 8 weeks after the surgery—the OGGT time profiles in individual dietary groups differed significantly depending on the type of surgery (Table 2). 

**Figure 2:**
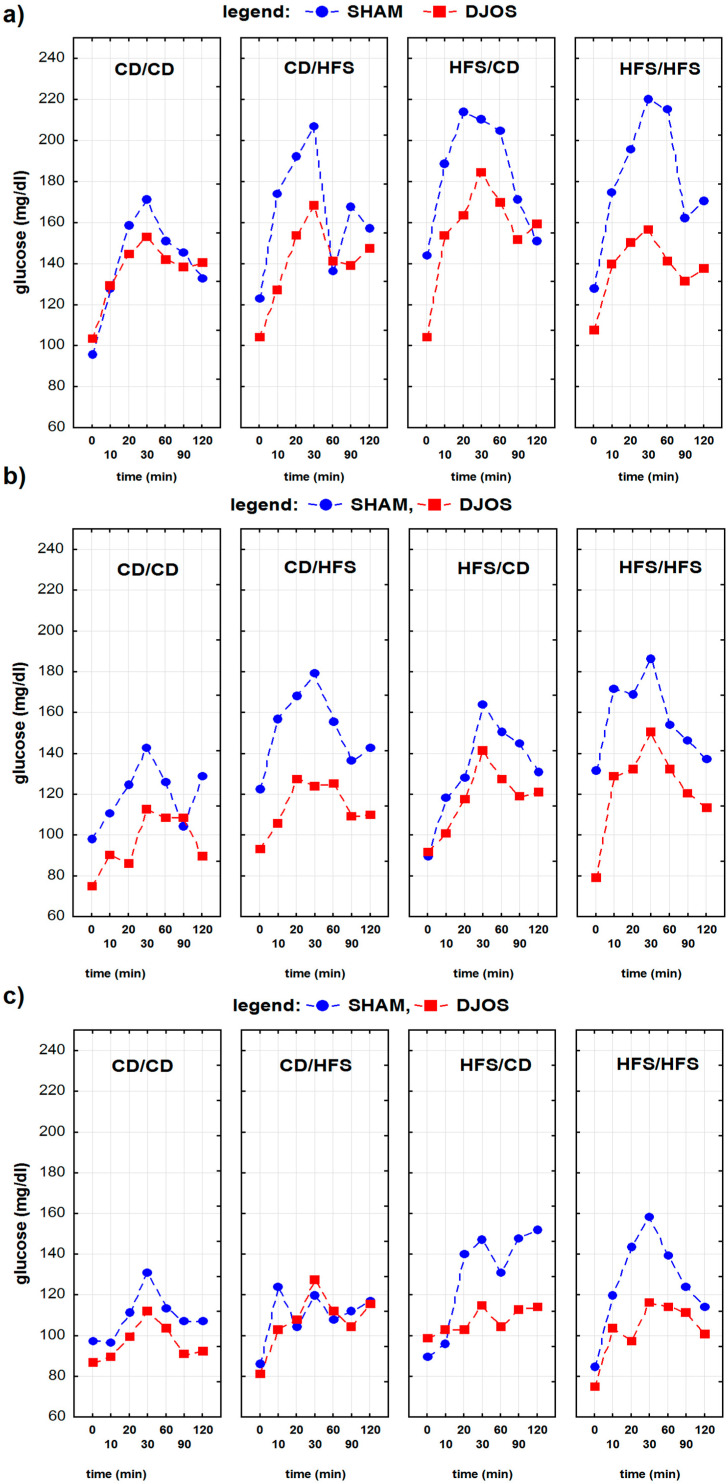
The oral glucose tolerance test (OGTT) results of Sprague–Dawley rats subjected to control (SHAM) and duodenojejunal omega switch (DJOS) surgery and different dietary regimes: (**a**) 8 weeks before the surgery, (**b**) 4 weeks after the surgery, (**c**) 8 weeks after the surgery. Legend: CD—control diet, HFS—high fat, high sugar diet, CD/CD—rats fed with CD diet before and after surgery, CD/HFS—rats fed with CD diet before but with HSF diet after the surgery, HFS/CD—rats fed with HFS diet before but CD diet after the surgery, HFS/HFS—rats fed with HFS diet before and after the surgery.

For the first OGTT, performed 8 weeks before the surgery, we found statistically significant differences in glucose concentration-time profiles between SHAM and DJOS-operated rats from the CD/HFS, HFS/CD, and HFS/HFS dietary groups (Table 3, Figure 2a). For the second OGGT, performed 4 weeks after the surgery, we found statistically significant differences between SHAM and DJOS-operated rats from the CD/CD, CD/HFS, and HFS/HFS dietary groups (Table 3). We observed higher glucose concentrations in SHAM-operated rats than in DJOS-operated animals’ treatment (Table 1, Figure 2b). The last OGTT, performed 8 weeks after the surgery, revealed the statistically significant differences between the time profiles of glucose concentrations for HFS/CD and HFS/HFS dietary groups (Table 3). The overall results of glucose concentrations during the OGTT were higher for SHAM-operated rats than for rats after DJOS surgery (Table 1, Figure 2c). 

### 3.2. Area under the Curve of the Oral Glucose Tolerance Test (AUC_OGTT_)

We found a statistically significant effect of the type of diet used on the AUC_OGTT_ value, regardless of the type of surgery performed. The lowest values of the AUC_OGTT_ were noted for the CD/CD groups for all three OGTT tests performed: 8 weeks before the surgery and 4 and 8 weeks after the surgery. We also found a statistically significant influence of the type of surgery on the AUC_OGTT_. In all the cases, the AUC_OGTT_ value was higher for the SHAM-operated rats than for the DJOS-operated ones. We found no statistically significant variances in the AUC_OGTT_ value when analyzing the interaction between the type of surgery performed and the type of diet used (Table 4).

At 8 weeks before the surgery, the values of the AUC_OGTT_ between SHAM and DJOS-operated rats from the CD/CD, CD/HFS, and HFS/CD dietary groups did not differ statistically. In contrast, such a difference was noted for the HFS/HFS dietary group (Table 4). At 4 weeks after the surgery, we noted no statistical differences in the AUC_OGTT_ values of SHAM and DJOS-operated rats only for rats fed with the control diet before and after the surgery (CD/CD). For the CD/HFS and HFS/HFS dietary groups, the differences in the AUC_OGTT_ values of the SHAM and DJOS-operated rats were significant. For the HFS/CD dietary group, the result of the contrast analysis of the AUC_OGTT_ values was at the limit of statistical significance (*p* = 0.056) (Table 4). In all cases, the AUC_OGTT_ values were lower in the DJOS-operated animals (Table 1). 

At 8 weeks after the surgery, we found no differences between the SHAM and DJOS-operated rats in the AUC_OGTT_ values for the CD/CD and CD/HFS dietary groups. In contrast, such differences existed for the HFS/CD and HFS/HFS dietary groups. Additionally, the value of the AUC_OGTT_ was smaller for rats after DJOS surgery (Table 1 and Table 4).

### 3.3. Hepatokines Plasma Concentrations

#### 3.3.1. Fetuin-B

Eight weeks after the surgery, the plasma concentrations of fetuin-B depended on the type of diet used in the experiment, type of surgery performed, and interaction between the diet and surgery (Table 5 and Table 6). Depending on the type of surgery performed (DJOS or SHAM), we observed the significant differences in fetuin-B concentration in the plasma of rats fed with the CD diet before the surgery (i.e., CD/CD, CD/HFS) (Table 6). Fetuin-B concentrations were lower in rats subjected to DJOS surgery when compared to those subjected to SHAM surgery (Table 5). In DJOS-operated rats, the highest fetuin-B plasma concentrations were found for the HFS/HFS and HFS/CD dietary groups. At the same time, the lowest values were noted for rats fed with the CD diet during the whole time of the experiment (CD/CD) (Table 5). Feeding the DJOS rats with the HFS diet, irrespective of the stage of the experiment, significantly increased the fetuin-B plasma concentration compared to the groups fed with the CD diet (Table 6). In SHAM-operated groups, higher levels of fetuin-B were observed in the plasma of rats fed with the CD diet before the surgery (CD/CD, CD/HFS) (Table 5 and Table 6).

#### 3.3.2. Growth Differentiation Factor-15 (GDF-15)

Eight weeks after the surgery, the GDF-15 plasma concentration depended on the type of the diet used and surgery performed, but not on the interaction between the diet and surgery (Table 5). We observed statistically significant differences between DJOS and SHAM-operated rats fed with HFS diet before the surgery (HFS/CD and HFS/HFS) (Table 5 and Table 6). We noted that the plasma GDF15 concentrations are slightly lower in rats subjected to DJOS surgery than in rats after SHAM surgery. The highest concentrations of GDF15, which differed significantly from the other groups, were noted for HFS/HFS dietary groups, both for DJOS and SHAM-operated rats. Additionally, the lowest concentration of plasma GDF15 was detected in rats from the CD/CD dietary group. We found that it was significantly different from the HFS/CD and HFS/HFS dietary groups (*p* < 0.001 for both DJOS and SHAM-operated rats from the HFS/CD groups, and *p* < 0.001 for the HFS/HFS group) (Table 5 and Table 6).

#### 3.3.3. Pentraxin 3 (PTX3)

Eight weeks after the surgery, the PTX3 plasma concentration depended on the type of diet used during the experiment and the interaction between the diet and the type of surgery (Table 5). We found statistically significant differences for the CD/CD and CD/HFS dietary groups when comparing DJOS and SHAM-operated arts (Table 6). In the rats fed with CD diet before and after the surgery, we observed higher levels of PTX3 in rats of the SHAM-operated group. We observed no significant differences in PTX 3 levels between DJOS-operated rats fed with different dietary patterns (Table 6). The PTX3 plasma levels were stable in this group, with negligible variances (Table 5 and Table 6). However, in the SHAM-operated rats, we observed statistically significant differences between the CD/CD and other dietary groups: CD/HFS, HFS/CD, and HFS/HFS (Table 6). We also observed that both groups of rats fed with the HFS diet after control surgery (CD/HFS and HFS/HFS) differed significantly from each other (Table 6). 

## 4. Discussion

In this study, we present the effects of duodenojejunal omega switch (DJOS) bariatric procedure on glucose tolerance and hepatokines (fetuin-B, growth differentiation factor-15, and pentraxin 3) levels in the plasma of Sprague–Dawley rats kept on different dietary patterns: CD/CD (control diet before and after the surgery), HFS/HFS (high fat, high sugar diet before and after the surgery), CD/HFS or HFS/CD (diet changing to the other one after the surgery). We report that (1) DJOS surgery had a beneficial effect on oral glucose tolerance test (OGTT) results and the area under the curve (AUC_OGTT_) values in all studied groups when compared to SHAM-operated rats; (2) the results of OGTT run at eight weeks before the surgery depended on the time regardless of the type of diet used and surgery performed (3) the results of the OGTT run at four and eight weeks after the surgery depending on the time elapsed, the diet applied, and the type of surgery performed; (4) the type of diet used in the experiment influenced all three analyzed hepatokines, (5) the type of surgery influenced fetuin-B and GDF15 plasma levels; (6) interaction between type of surgery performed and diet used influenced the concentration of fetuin-B and pentraxin 3.

Our study showed that DJOS bariatric surgery helps regulate glucose metabolism even in the presence of a deleterious type of high fat, high sugar diet, which is associated with inflammation, severe steatosis, oxidative stress, and insulin resistance. DJOS-operated rats exhibited significantly reduced AUC_OGTT_ values in all studied dietary groups. This result agrees with our previous study showing significant improvement in the glucose tolerance measured by OGTT in DJOS-operated Sprague–Dawley rats maintained on HF/HF (high fat/high fat) and CD/CD dietary regime. For those groups, the 30- and 60-min plasma glucose levels were consistently lower compared to SHAM-operated rats [22]. Other researchers presented similar results. Cummings et al. [23] reported a 17% reduction in AUC_OGTT_ one month after ileal transposition in the UCD-T2DM rat [23]. 

Our study showed that both the diet and type of surgery and the combination of those two factors affected fetuin-B levels. We observed that fetuin-B plasma concentrations were considerably higher in rats fed with high fat and high carbohydrate diet before the DJOS surgery than those fed with the control diet. This finding corresponds to the results obtained by Choi et al. [24] who also reported that fetuin-B and zinc-α2-glycoprotein plasma levels were significantly elevated in obesity-resistant rats exposed to a high-fat diet. Moreover, in our study, the DJOS-operated rats fed with the control diet before the surgery presented much lower fetuin-B concentrations than rats fed with the HFS diet. The HFS diet used before DJOS surgery significantly lowered DJOS efficiency reflected by fetuin-B levels. Our results may suggest that an early-introduced proper feeding regime positively affects carbohydrate metabolism later in life. Qu et al. [25], in the study conducted on mice, showed a positive dependence of fetuin-B, HbA1c, and HOMA-IR and their correlation with obesity and impaired lipid metabolism prevalence.

The other fetuin, fetuin-A, is positively associated with HbA1c and fasting blood glucose, thus is described as an independent risk factor for *diabetes mellitus* development. The people with impaired glucose tolerance showed elevated plasma concentration of fetuin-A, independent of NAFLD as comorbidity [26]. Khadir et al. [26], in their study, noted that adipocytes are an important source of fetuin-A Considering the similarities in the fetuin-A and fetuin-B structure, we conclude that it would be beneficial to evaluate fetuin-B activity in the adipose tissue. The glucose metabolism regulated by fetuin-A seems to act primarily via insulin pathways signaling. The fetuin-B mode of interaction is still a matter of further investigation, although it may be connected to glucose effectiveness [27]. Although we did not focus on this aspect, we showed that rats fed with the HFS diet showed considerably higher fetuin-B plasma concentrations eight weeks after the surgery and 16 weeks into the experiment. Peter et al. [27], in the study conducted on human subjects, proved that fetuin-B levels correlated positively with the glucose AUC_OGTT_. Their result supported the observation that fetuin-B may be involved in regulating glucose effectiveness, which refers to glucose ability to promote its disposal, independently of insulin [27]. When analyzing our OGTT results, we can see that the glycemia is also much higher in rats fed with the HSF diet before or after the DJOS surgery. Mokou et al. [28], in their study on women with polycystic ovarian syndrome (PCOS), showed elevated levels of fetuin-B in the serum of the studied subjects. Moreover, they found that fetuin-B concentration in the serum was even higher in obese subjects when compared with those without this comorbidity. Therefore, they suggested that fetuin-B might be associated with indicators of lipid and glucose metabolism and markers of inflammation [28]. On the other hand, Xing et al. [29] reported that the elevated levels of fetuin-B could be at least partly associated with the higher risk of myocardium ischemia. According to the authors, it acts via insulin receptors since the results showed that higher fetuin-B concentrations could exacerbate reperfusion injuries after ischemia in patients with T2DM [29].

Our study showed that both the diet and the type of surgery affect the growth differentiation factor-15 (GDF-15) level independently. The lowest GDF-15 concentration was observed in rats fed with the CD diet after SHAM and DJOS surgery. While the highest GDF-15 level was noted in rats fed with the HFS diet—both DJOS and SHAM and they simultaneously presented the highest glycemia. This observation may confirm the existing reports saying that GDF-15 levels are elevated in patients with impaired glucose tolerance [30]. Baek et al. [31] even suggested that the elevated level of GDF-15, resulting from weight loss and reduced fat intake, could become a target for T2DM treatment. Our results showed that DJOS-operated rats presented significantly higher GDF-15 levels than SHAM-operated rats. Our observation is consistent with the results of Kleinert et al. [32], who investigated the effects of Roux-en-Y gastric bypass (RYGB) surgery in obese patients with normal glucose tolerance and T2DM. They observed that the increase in GDF-15 levels manifested earlier than the body weight loss, and the increase was proportional to the weight loss [32]. Although Kleinert et al. study [32] and ours report on the effects of different bariatric surgeries, the mechanism of action of these two procedures is very similar. Both in DJOS and RYGB, a fragment of the intestine is excluded from the intestinal passage, which is crucial for the effect. According to the hindgut hypothesis, such omission allows for faster food passage to the hindgut. This change leads to a rapid increase in GLP-1 levels that limit further nutrients absorption and increase insulin production. GDF-15 levels are influenced by multiple factors, such as stress, surgery, pharmacology, aging, and glycemia. Kleinert et al. suggested that it is imperative to not look at GDF-15 in isolation but to follow GDF-15 over time and carefully characterize the study subjects to get a better picture of human GD-F15 physiology [32].

Zempo-Miyaki et al. [17] showed that weight reduction observed after lifestyle modification can increase PTX3 levels in overweight and obese men. They also showed that habitual exercise could increase PTX3 levels in patients achieving normal weight (BMI < 25 kg/m^2^) [17]. Other studies on obese pre-menopause women showed that plasma PTX3 levels significantly increased after dramatic weight loss achieved with dietary modification, aerobic exercise training, and novel resistance training [33]. Slusher and Huang [34] reported the increased plasma PTX3 concentrations in obese individuals after acute aerobic exercise, which depended on the patients’ maximal oxygen uptake capacity [34]. Studies on bariatric surgery effects showed that gastric banding could increase plasma PTX3 concentrations in morbidly obese patients six months or one year after weight reduction [35]. The results of two separate experiments, where we used HF [22] or HFS [36] diets, showed that a diet change after DJOS surgery to a more caloric one, regardless if it was HF or HFS, did not lead to significant weight loss [22,36]. In our previous separate study on this animal model, we observed that a high fat diet reduced the effects of the surgery compared to groups of rats fed with the control diet [22]. 

Here, we used the HFS diet and we observed that SHAM-operated rats from the CD/CD dietary group presented the highest concentration of PTX3. Other combinations of the diet reduced PTX3 levels in the plasma of SHAM-operated rats. We have not observed significant changes in PTX3 plasma level of DJOS-operated rats in the groups with the diet changed after surgery. That may suggest that changes in the dietary protocol before and after DJOS surgery change the physiological and biochemical pathways and lead to metabolic influence PTX3 levels compared to SHAM-operated rats. Together with insignificant bodyweight reduction in the DJOS-operated rats, described in the previous study [36], we postulate that bariatric protocol does not reduce deleterious effects of the HFS/HFS diet. The postulate can be supported by no significant changes in PTX3 concentration observed in DJOS-operated rats. 

## 5. Conclusions

Duodenojejunal omega switch (DJOS) surgery helps regulate the metabolic conditions of Sprague–Dawley DIO rats fed with HFS combined with CD dietary regimes. The type of diet used before and after DJOS bariatric surgery had a more significant impact on selected hepatokines levels than the DJOS protocol itself. DJOS types of bariatric surgery showed a positive impact on fetuin-B, growth differentiation factor-15 (GDF-15) leading to its reduction, but not pentraxin 3 (PTX3) in rats fed with the HFS diet. We conclude that a dietary regime is essential to achieve therapeutic and clinical goals after bariatric surgery.

## Figures and Tables

**Table 1:** Time profile of glucose concentration (mg/dl) and area under the curve (AUC) of the oral glucose tolerance test (OGTT) of Sprague–Dawley rats subjected to control (SHAM) and duodenojejunal omega switch (DJOS) surgery and different dietary regimes at 8 weeks before and 4 and 8 weeks after the surgery. The results are presented as mean ± standard deviation.

Time of OGTT	Time after Glucose Administration [min]	SHAM-Operated Groups				DJOS-Operated Groups			
		CD/CD	CD/HFS	HFS/CD	HFS/HFS	CD/CD	CD/HFS	HFS/CD	HFS/HFS
8 weeks before surgery	0	95 ± 8	123 ± 17	144 ± 23	128 ± 18	103 ± 14	104 ± 9	104 ± 18	108 ± 22
	10	128 ± 14	174 ± 16	189 ± 25	175 ± 33	129 ± 5	127 ±13	154 ± 28	140 ± 11
	20	159 ± 8	192 ± 23	214 ± 38	195 ± 37	144 ± 3	154 ±17	164 ± 29	150 ± 15
	30	171 ± 3	207 ± 20	210 ± 33	220 ± 47	153 ± 8	168 ± 21	184 ± 36	156 ± 16
	60	151 ± 19	136 ± 60	205 ± 41	215 ± 57	142 ± 10	141 ± 28	169 ± 34	141 ± 31
	90	145 ± 13	168 ± 25	171 ± 17	162 ± 24	138 ± 23	139 ± 20	151 ± 22	132 ± 15
	120	133 ± 10	157 ± 38	151 ± 38	170 ± 10	140 ± 23	147 ± 11	159 ± 32	138 ± 31
	AUC	400,018 ± 46,876	436,264 ± 194,926	672,600 ± 216,520	734,562 ± 262,214	339,529 ± 38,905	369,225 ± 90,497	496,578 ± 191,264	348,584 ± 106,791
4 weeks after surgery	0	98 ± 18	122 ± 7	90 ± 4	132 ± 30	75 ± 4	93 ± 13	92 ± 12	79 ± 14
	10	110 ± 8	157 ± 18	118 ± 13	171 ± 42	90 ± 10	106 ± 5	101 ± 4	129 ± 23
	20	125 ± 8	168 ± 17	128 ± 14	169 ± 32	86 ± 18	128 ± 14	118 ± 7	132 ± 18
	30	143 ± 11	179 ± 33	164 ± 32	186 ± 33	113 ± 13	124 ± 6	142 ± 8	150 ± 26
	60	126 ± 17	156 ± 14	151 ± 25	154 ± 24	108 ± 2	125 ± 11	127 ± 12	133 ± 43
	90	104 ± 15	136 ± 15	145 ± 30	146 ± 30	108 ±12	109 ± 10	119 ± 12	121 ± 47
	120	129 ± 11	143 ± 14	131 ± 31	137 ± 28	90 ± 9	110 ± 16	121 ± 26	113 ± 36
	AUC	14,533 ± 410	18,341 ± 2056	17,014 ± 1692	18,829 ± 3069	12,227 ± 554	13,937 ± 455	14,668 ± 1010	15,294 ± 3849
8 weeks after surgery	0	97 ± 6	86 ± 10	90 ± 7	85 ± 5	87 ± 6	81 ± 10	99 ± 5	75 ± 13
	10	96 ± 12	124 ±1	96 ± 15	120 ± 12	89 ± 6	103 ± 13	103 ± 7	104 ± 13
	20	112 ± 16	104 ± 5	140 ± 34	144 ± 14	99 ± 8	108 ± 11	103 ± 8	97 ± 5
	30	131 ± 15	120 ± 6	147 ± 38	158 ± 14	112 ± 8	127 ± 15	115 ± 10	116 ± 17
	60	113 ± 25	108 ± 8	131 ± 36	139 ± 14	104 ± 23	112 ± 4	104 ± 9	114 ± 6
	90	107 ± 31	112 ± 7	148 ± 35	124 ± 20	91 ± 17	104 ± 9	113 ± 8	111 ± 9
	120	107 ± 23	117 ± 9	152 ± 37	114 ± 19	92 ± 17	116 ± 3	114 ± 4	101 ± 16
	AUC	13,390 ± 2209	13,433 ± 600	16,395 ± 3693	15,815 ± 1349	11,784 ± 1547	13,279 ± 274	13,067 ± 791	12,980 ± 913

**Table 2:** Comparison of the oral glucose tolerance test (OGTT) results of Sprague–Dawley rats subjected to control (SHAM) and duodenojejunal omega switch (DJOS) surgery and different dietary regimes in terms of the time of glucose administration (p_time_), dietary pattern and time (p_time×diet_), and interaction between those parameters (p_time×diet×surgery_). Statistical significance was set at *p* < 0.05.

Time of OGTT	p_time_	p_time×diet_	p_time×diet×surgery_
8 weeks before surgery	<0.001	0.161	0.114 *
4 weeks after surgery	<0.001	<0.001	<0.05
8 weeks after surgery	<0.001	<0.001	<0.01

Legend: * This result does not present the interaction with the surgery, but the assignment to the study group.

**Table 3:** Comparison of glucose concentration changes in time during the oral glucose tolerance test (OGTT) in Sprague–Dawley rats subjected to control (SHAM) surgery and different dietary regimes and Sprague–Dawley rats subjected to duodenojejunal omega switch (DJOS) surgery and different dietary regimes.

Time of OGTT	p_SHAM vs. DJOS_			
	CD/CD	CD/HFS	HFS/CD	HFS/HFS
8 weeks before surgery	0.601	<0.01	<0.001	<0.001
4 weeks after surgery	<0.05	<0.001	0.086	<0.001
8 weeks after surgery	0.073	0.700	<0.01	<0.01

Legend: *p*—significance level.

**Table 4:** Results of contrast analysis of the area under curve (AUC) of the oral glucose tolerance test (OGTT) of Sprague–Dawley rats subjected to control (SHAM) and duodenojejunal omega switch (DJOS) surgery and different dietary regimes at 8 weeks before (AUC 1) and 4 weeks (AUC 2) and 8 weeks (AUC 3) after the surgery. Statistical significance was set at *p* < 0.05.

Time of OGTT	p_diet_	p_surgery_	p_diet×surgery_
AUC 1	<0.001	<0.01	0.067
AUC 2	<0.01	<0.001	0.568
AUC 3	<0.05	<0.001	0.136
**Dietary group**	**p_SHAM vs. DJOS_**		
	**AUC 1**	**AUC 2**	**AUC 3**
CD/CD	0.527	0.069	0.121
CD/HFS	0.483	<0.001	0.880
HFS/CD	0.061	0.056	<0.01
HFS/HFS	<0.001	<0.01	<0.01

**Table 5:** Fetuin-B, growth differentiation factor-15 (GDF-15), and pentraxin 3 concentrations in plasma of Sprague–Dawley rats subjected to control (SHAM) and duodenojejunal omega switch (DJOS) surgery and different dietary regimes eight weeks after the surgery. The results are presented as mean ± standard deviation. Statistical significance was set at *p* < 0.05.

Hepatokine Concentration	Surgery Group	Dietary Groups				p_ANOVA_		
		CD/CD	CD/HFS	HFS/CD	HFS/HFS	Diet	Surgery	Diet × Surgery
Fetuin-B [ng/mL]	SHAM	359.6 ± 16.3	349.9 ± 16.7	301.2 ± 40.2	314.1 ± 6.6	<0.001	<0.001	<0.001
	DJOS	180.7 ± 66.1	257.7 ± 45.5	302.4 ± 15.3	302.8 ± 15.6			
GDF-15 [ng/mL]	SHAM	128.6 ± 36.6	144.9 ± 22.0	179.7 ± 33.1	269.8 ± 64.7	<0.001	<0.01	0.728
	DJOS	90.5 ± 37.7	129.4 ± 17.1	134.9 ± 23.6	227.9 ± 19.4			
Pentraxin 3 [pg/mL]	SHAM	929.7 ± 37.1	817.4 ± 73.6	858.8 ± 42.5	881.6 ± 8.6	<0.01	0.200	<0.01
	DJOS	884.6 ± 1.5	882.2 ±13.2	882.7 ± 5.7	888.2 ± 1.8			

**Table 6:** Contrast analysis Fetuin-B, growth differentiation factor-15 (GDF-15), and pentraxin 3 concentrations in plasma of Sprague–Dawley rats subjected to control (SHAM) and duodenojejunal omega switch (DJOS) surgery and different dietary regimes eight weeks after the surgery. Statistical significance was set at *p* < 0.05.

Hepatokine Concentration	Surgery Group	p_CD/CD vs. CD/HFS_	p_CD/CD vs. HFS/CD_	p_CD/CD vs. HFS/HFS_	p_CD/HFS vs. HFS/CD_	p_CD/HFS vs. HFS/HFS_	p_HFS/CD vs. HFS/HFS_	p_SHAM vs. DJOS_			
								CD/CD	CD/HFS	HFS/CD	HFS/HFS
Fetuin-B [ng/mL]	SHAM	0.620	<0.01	<0.05	<0.05	0.071	0.508	<0.001	<0.001	0.933	0.058
	DJOS	<0.001	<0.001	<0.001	<0.05	<0.001	<0.05				
GDF-15 [ng/mL]	SHAM	0.423	<0.05	<0.001	0.092	<0.001	<0.001	0.066	0.446	<0.05	<0.05
	DJOS	0.061	<0.05	<0.001	0.783	<0.001	<0.001				
Pentraxin 3 [pg/mL]	SHAM	<0.001	<0.001	<0.05	<0.05	<0.01	0.243	<0.05	<0.01	0.222	0.731
	DJOS	0.902	0.920	0.850	0.982	0.756	0.773				

## Data Availability

The data are available after contact with the corresponding author.

## Abbreviations

AUC—area under the curve, CD—control diet, HFS—high fat, high sugar diet, CD/CD—rats fed with CD diet before and after surgery, CD/HFS—rats fed with CD diet before but with HSF diet after the surgery, DJOS—duodenojejunal omega switch, HFS/CD—rats fed with HFS diet before but CD diet after the surgery, HFS/HFS—rats fed with HFS diet before and after the surgery, SHAM—control surgery. OGTT—oral glucose tolerance test.
